# Dysregulation of Hypothalamic Gene Expression and the Oxytocinergic System by Soybean Oil Diets in Male Mice

**DOI:** 10.1210/endocr/bqz044

**Published:** 2020-01-08

**Authors:** Poonamjot Deol, Elena Kozlova, Matthew Valdez, Catherine Ho, Ei-Wen Yang, Holly Richardson, Gwendolyn Gonzalez, Edward Truong, Jack Reid, Joseph Valdez, Jonathan R Deans, Jose Martinez-Lomeli, Jane R Evans, Tao Jiang, Frances M Sladek, Margarita C Curras-Collazo

**Affiliations:** 1 Department of Molecular, Cell and Systems Biology, University of California, Riverside, California; 2 Neuroscience Graduate Program, University of California, Riverside, California; 3 Department of Computer Science and Engineering, University of California Riverside, California

**Keywords:** oxytocin, coconut oil, Plenish, diabetes, linoleic acid, stigmasterol, high-fat diet

## Abstract

Soybean oil consumption has increased greatly in the past half-century and is linked to obesity and diabetes. To test the hypothesis that soybean oil diet alters hypothalamic gene expression in conjunction with metabolic phenotype, we performed RNA sequencing analysis using male mice fed isocaloric, high-fat diets based on conventional soybean oil (high in linoleic acid, LA), a genetically modified, low-LA soybean oil (Plenish), and coconut oil (high in saturated fat, containing no LA). The 2 soybean oil diets had similar but nonidentical effects on the hypothalamic transcriptome, whereas the coconut oil diet had a negligible effect compared to a low-fat control diet. Dysregulated genes were associated with inflammation, neuroendocrine, neurochemical, and insulin signaling. *Oxt* was the only gene with metabolic, inflammation, and neurological relevance upregulated by both soybean oil diets compared to both control diets. Oxytocin immunoreactivity in the supraoptic and paraventricular nuclei of the hypothalamus was reduced, whereas plasma oxytocin and hypothalamic *Oxt* were increased. These central and peripheral effects of soybean oil diets were correlated with glucose intolerance but not body weight. Alterations in hypothalamic *Oxt* and plasma oxytocin were not observed in the coconut oil diet enriched in stigmasterol, a phytosterol found in soybean oil. We postulate that neither stigmasterol nor LA is responsible for effects of soybean oil diets on oxytocin and that *Oxt* messenger RNA levels could be associated with the diabetic state. Given the ubiquitous presence of soybean oil in the American diet, its observed effects on hypothalamic gene expression could have important public health ramifications.

The hypothalamus is part of a complex network in the brain that is responsible for sensing nutritional status and executing behavioral and metabolic responses to changes in fuel availability. As part of this network, the hypothalamus produces intrinsic peptides and neurotransmitters that influence food intake, energy balance, and glucose homeostasis (1, 2). One such hypothalamic anorexigenic peptide is oxytocin (OXT), which is synthesized in the magnocellular neuroendocrine cells (MNCs) and acts as a peripheral hormone after being released from axons in the posterior pituitary into the systemic circulation. Oxytocin is also released centrally from the soma and dendrites of MNCs within the paraventricular (PVN) and supraoptic (SON) nuclei of the hypothalamus (3, 4). Another component of the central oxytocinergic signaling is the oxytocin-synthesizing parvocellular neurons of the PVN that project to autonomic brainstem regions and likely mediate leptin anorectic signals (2). Expression of *Oxt* and the related neuropeptide, vasopressin (*Avp*), is increased both in induced and spontaneous models of diabetes (5). Centrally, oxytocin is involved in feeding regulation and energy expenditure. For example, mice lacking OXT or its receptor exhibit mild or late-onset obesity, although normal body weight has also been reported (6).

It has been shown that energy-dense, high-fat diets (HFD) disrupt energy balance and cause changes in body weight regulation and an upregulation of proinflammatory cytokines and insulin resistance in the hypothalamus and other tissues (7-9). Although it is clear that the hypothalamus is a key player in controlling the balance between energy homeostasis and obesity/diabetes, the specific mechanisms regulating that balance remain elusive. Even less well-studied is the impact of specific components of the diet on the hypothalamus.

Individual risk factors such as the consumption of fat-rich foods and a sedentary lifestyle are thought to contribute significantly to the rapid rise in the prevalence of obesity and comorbid diseases such as diabetes (10, 11). Whereas the vast majority of diet-induced obesity studies focus on the role of saturated fats, such as those found in animal fat, a growing body of evidence suggests that polyunsaturated fatty acids (PUFAs), such as those found in vegetable oils, also contribute to the obesity epidemic (12-17). For example, there has been a 1000-fold increase in the consumption of soybean oil in the United States during the 20th century and, as a result, the per capita consumption of its primary unsaturated fatty acid component, linoleic acid (LA, C18:2), has increased from less than 1% to approximately 7.4% of energy intake (18). Soybean (SO) and other oils high in LA have been shown by us and others (12-14, 16) to be obesogenic and diabetogenic in rodent systems, and we have shown that a diet enriched in SO similar to the American diet causes a global dysregulation of hundreds of genes in the liver compared to an isocaloric coconut oil (CO) diet (14).

Although neither our study, nor any other we could find in the literature, has examined the impact of a soybean oil diet on the hypothalamic transcriptome, alterations in dietary PUFAs such as LA and omega-3 fatty acids and their metabolites have been shown to modulate fatty acid levels in different regions of the brain, including the hypothalamus (19, 20). Unsaturated fats have also been shown to affect food intake, glucose homeostasis, and gene expression in the brain (21-24). In particular, LA has been proposed to target specific orexigenic (*Agrp*) and anorexigenic (POMC, Cart) neuronal populations in the hypothalamus (16) and to increase levels of hypothalamic arachidonic acid, a potent proinflammatory molecule (25). Thus, the obesogenic and diabetogenic properties of an SO-based diet may be due to modulation of proinflammatory processes and/or hypothalamic signaling of key neuropeptide populations by LA.

Non–fatty acid components in SO include phytosterols such as stigmasterol (ST), campesterol, and β-sitosterol. Of these, ST has been shown to have metabolic effects such as increasing insulin synthesis and secretion and causing cholesterol efflux in macrophage foam cells (26, 27). The metabolic effects of ST may be due to its role as a ligand for 2 nuclear receptors: It is an agonist for the liver X receptor (LXR, *Nr1h3*) (28, 29), which is expressed in the hypothalamic nuclei, and an antagonist for the farnesoid X receptor (FXR, *Nr1h4*) (30). Because both nuclear receptors are linked to food intake, energy expenditure, and glucose and lipid homeostasis (31, 32), ST may influence the development of obesity and/or diabetes by a diet enriched in SO.

In this study, we hypothesized that an SO-rich diet, such as that currently consumed in the United States, affects hypothalamic gene expression and oxytocin peptide levels differentially from other HFDs, that those changes correlate with obesity and/or diabetes, and that they involve one or both of its major components, LA and ST. To test these hypotheses, we subjected male C57BL/6N mice to 4 isocaloric HFDs composed of CO (largely medium-chain saturated fats), conventional SO (high in LA), a genetically modified SO low in LA (Plenish, PL; DuPont Pioneer) or CO supplemented with ST. We performed RNA-sequencing (RNA-seq) analysis on the hypothalamus of the mice fed the first 3 diets (and a low-fat control diet) and analyzed hypothalamic and peripheral levels of oxytocin in mice fed all 4 diets. The results show that compared to the CO and low-fat control diets, the 2 SO-based diets resulted in a significant dysregulation of more than 100 hypothalamic genes including those involved in neurochemical and neuroendocrine pathways and metabolic and neurological disorders. With only 2 exceptions, the changes in gene expression were not significantly different between the high-LA and low-LA SO diets. Importantly, oxytocin was significantly upregulated on both the RNA level in the hypothalamus and the peptide level in the plasma in both SO diets, but not the CO diet. In contrast, immunohistochemical (IHC) staining of the SON and PVN in the hypothalamus showed reduced OXT protein levels in both sets of SO-fed mice, consistent with increased peripheral release of OXT hormone. The ST diet did not affect hypothalamic oxytocin expression or plasma levels nor promote obesity or diabetes compared to the CO diet. Taken together, our results support the hypothesis that an SO-enriched diet affects gene expression in the hypothalamus, including *Oxt,* and causes an elevated level of circulating oxytocin. Although these effects correlated significantly with diabetes as measured by a glucose tolerance test (GTT), they did not correlate with body weight nor seem to involve either LA or ST.

## Methods

### Diets

Three isocaloric diets with 40 kcal% fat (4.87 kcal/gm) (Table 1) were formulated in conjunction with Research Diets, Inc. The diets are based on the Surwit diet, which is widely used in diet-induced obesity studies and formulated with elements from the AIN-93 diet. The 5% fiber from cellulose in the AIN diet is replaced with cornstarch. These 40 kcal% diets have been described previously (13, 14) and include 1) CO providing 36 kcal% from CO and 4 kcal% from conventional SO to provide the essential fatty acids LA and α-LA; 2) SO + CO providing 21 kcal% fat calories from CO and 19 kcal% from SO, of which 10 kcal% are from LA; 3) PL + CO in which conventional SO was replaced on a per-gram basis with a genetically modified high oleic oil, Plenish (PL; DuPont Pioneer). A fourth diet (ST + CO) contained 40 kcal% fat from CO plus 0.1 g ST (Sigma-Aldrich S2424), equivalent to the amount that a 40 kcal% SO diet would contain. To determine the contribution of LA to our observed phenotype, we chose a control fat that is plant based but high in saturated fat and low in LA, ie, CO. Furthermore, to make the diets isocaloric without increasing the LA content in SO + CO, we chose to add some CO to our other 2 HFDs. This design allowed us to test the effect of saturated fat without LA (CO) vs unsaturated fats with different levels of LA. SO + CO had higher levels of LA than PL + CO, thus allowing us to elucidate the effect of LA. CO vs PL + CO allowed investigation of the effect of saturated vs unsaturated fats. The fatty acid (Table 2) and phytosterol (Table 3) composition of PL, conventional SO, and CO used in the diets were determined by Covance Laboratories. The total amount of carbohydrates and protein were constant across all diets, including the low-fat control: vivarium chow (Viv) (Purina Test Diet 5001, Newco Distributors) with 3.36 kcal/g fat. Viv chow also contains approximately 25% fiber.

**Table 1.** Composition of diets and oils used in study

Nutrient Composition of Diets, gm %					
	Viv Chow^*a*^	CO	SO + CO	PL + CO	ST + CO
Protein	23.9	20.1	20.1	20.1	20.1
Carbohydrate	48.7	53.4	53.4	53.4	53.4
Fat	5	21.5	21.5	21.5	21.5
kcal/gm	3.36	4.87	4.87	4.87	4.87
Fat, kcal %	13.4	40	40	40	40
Source of fat, gm %	Viv Chow^*a*^	CO	SO + CO	PL + CO	ST + CO
Porcine animal fat	4.5	0	0	0	0
SO oil	0	25	115	0	25
PL oil	0	0	0	115	0
CO, hydrogenated	0	220	130	130	220
Sterols, gm/1.1 kg					
ST	0	0	0.1	0.1	0.1

Abbreviations: CO, coconut oil; PL, Plenish; SO, soybean oil; ST, stigmasterol.

^*a*^Purina Test Diet 5001.

**Table 2.** Fatty acid composition of oils used in study

Fatty Acid (%)	Coconut	Soybean	Plenish
Lauric (12:0)	45	< 0.05	< 0.05
Myristic (14:0)	17.5	0.07	< 0.05
Palmitic (16:0)	8.67	10.6	5.81
Stearic (18:0)	10.2	3.98	4.17
Oleic (18:1)	0.25	20.9	73.9
Linoleic (18:2ω6)	< 0.06	52.9	7.42
α-linolenic (18:3ω3)	< 0.06	6.54	1.91
ω6:ω3 (18:2/18:3)	0	8.1	3.4

Composition was determined by Covance Labs.

No isoflavones were detected in these oils.

**Table 3.** Phytosterol composition of oils used in study

Phytosterol, mg/100 gm	Coconut	Soybean	Plenish
Campesterol	6.6	61.1	61.1
Stigmasterol	9.1	65	64
β-sitosterol	44.9	177	131
Brassicasterol	< 1	< 1	< 1
Other sterols	24.7	31.6	23.9
Total sterols	85.3	335	280

Composition was determined by Covance Labs.

No isoflavones were detected in these oils.

Diets were provided to the animals in pellet form, twice weekly for up to 24 weeks; the amount of food consumed was calculated weekly on a per-cage basis. Food intake (in terms of total calories consumed) was not significantly different between any of the HFD groups. The Viv chow group consumed a greater quantity of food by mass because it has a much lower caloric density.

### Animals

Care and treatment of animals was in accordance with guidelines from and approved by the University of California, Riverside Institutional Animal Care and Use Committee (Animal Use Protocol No. 20140014 and No. 20140017). All mice had ad libitum access to food and water (other than the indicated fasting times). At the end of the study, some mice were humanely killed by CO_2_ inhalation and their brains snap-frozen. Others were subjected to tail blood draw or cardiac puncture and humanely killed by cervical dislocation, or transcardial perfusion under isoflurane anesthesia (see as follows), in accordance with stated National Institutes of Health guidelines.

**Figure 1.**
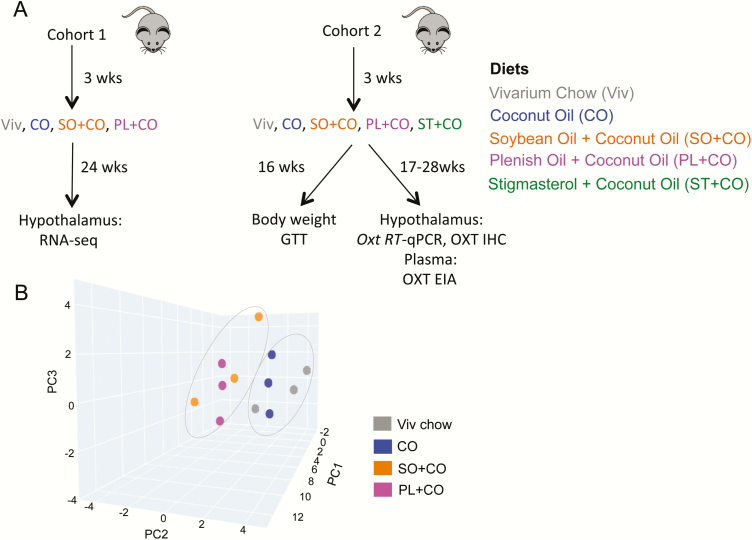
Study design and 3-dimensional principal components analysis (PCA) showing differential effects of dietary fat on hypothalamic gene expression. A, Workflow showing the 2 cohorts of mice used, different diets, time on diets, and various analyses performed. B, PCA of RNA-sequencing data from hypothalami of male mice fed Viv chow, CO, SO + CO or PL + CO for 24 weeks showing 3 biological replicates for each diet. As indicated, the 2 soybean oil diets, SO + CO and PL + CO, can be grouped together as well as Viv chow and CO. CO, coconut oil diet; EIA, enzyme immunoassay; GTT, glucose tolerance test; IHC, immunohistochemistry; RT-qPCR, reverse transcriptase-quantitative polymerase chain reaction; Viv chow, vivarium chow (low-fat control). CO diets enriched in conventional soybean oil (SO + CO), genetically modified Plenish (PL + CO), and stigmasterol (ST + CO).

Male C57BL/6N mice (Charles River Laboratories) were bred in-house and maintained on a 12:12–hour light-dark cycle in a specific pathogen-free vivarium for the 40 kcal% diet experiment (cohort 1) (Fig. 1A). Pups were weaned at age 3 weeks and started on 1 of the 4 different diets (Viv Chow, CO, SO + CO, PL + CO). At least 12 mice were placed on each diet, with 3 or 4 animals housed per cage.

Mice were humanely killed (by CO_2_ inhalation) at 24 weeks after weaning and their brains harvested and stored at –80°C for subsequent transcriptomic analysis. To isolate the hypothalamus, brains were slightly thawed in a dry-ice filled container and blocked manually at the anterior commissure rostrally, at mammillary bodies caudally (1 mm rostral to end of median eminence), and at the top of third ventricle dorsally, and then the entire hypothalamus was removed. Glucose tolerance, food consumption, and body weight data from these mice have been published previously (13).

Cohort 2 mice, housed in a conventional vivarium, were subjected to Viv chow, CO, SO + CO, PL + CO, or ST + CO diets starting at age 3 weeks and their tissues were used in immunoassay, IHC, and quantitative polymerase chain reaction (qPCR) experiments (Fig. 1A). Weekly change in body mass (expressed as body weight) per mouse was measured in grams for up to 16 weeks after the start of diets. Food consumed on a given diet was measured on a per-cage basis, normalized to the number of mice per cage. Food was changed and measured twice weekly; values were combined to generate the weekly average. Glycemia after fasting and glucose load (GTT) were also measured, as described previously (13, 14), after 16 weeks on the diets. Mice were humanely killed one to several weeks later. Some mice were subjected to cardiac puncture under isoflurane anesthesia for collection of blood for use in immunoassay experiments. Other mice were transcardially perfused for use in brain IHC experiments. For a subset of mice, death consisted of CO_2_ inhalation followed by decapitation for brains to be snap-frozen for use in qPCR experiments.

### Glucose tolerance test

GTT was performed as described previously (14). Briefly, mice were fasted overnight and then injected with D-glucose 2 g/kg body weight by an intraperitoneal injection of a sterile 20% glucose solution in 0.9% saline. Under light restraint tail blood was drawn and glycemia measured before (0 minutes) and after injection (at 15, 30, 60, and 120 minutes). Area under the curve was plotted for group comparisons.

### RNA extraction and sequencing

Total RNA was isolated from hypothalamic homogenates using an miRNeasy kit (Qiagen, Inc) and evaluated for purity and concentration by NanoDrop (NanoDrop Technologies) and for quality using Agilent 2100 Bioanalyzer. Poly(A)^+^ RNA (4 μg) having an RNA Integrity Number (RIN) of greater than 7.5 was used to construct sequencing libraries with the TruSeq Long RNA Sample Prep Kit (Illumina). RNA libraries were validated for RNA integrity by Bioanalyzer, pooled in equimolar amounts, and sequenced on an Illumina HiSeq 2000 at the UCR Genomics Core to generate 50 base, paired-end reads. Three biological replicates each for Viv Chow, CO, SO + CO, and PL + CO were sequenced yielding approximately 15 million reads per sample.

### Differential gene expression analysis using RNA sequencing data

Reads were aligned to the mouse reference genome (mm10) with TopHat v2.1.1 using the default parameters with the exception that only one unique alignment for a given read was allowed. Raw read counts were calculated at the gene level for each sample using HTSeq v0.6.1. Library normalization was performed with EDASeq (33); within-lane normalization on GC content was performed with the LOESS method and between-lane normalization was performed with a nonlinear full quantile method. Normalization factors from EDASeq were used for differential expression analysis with DESeq2. Normalized read counts, and fragments per kilobase per million and rlog (regularized log transformation) results were generated for downstream analysis. Heat maps were generated with the pheatmap package in R using rlog values. Genes that had a log2 fold-change greater than or equal to .5 and *P*adj less than or equal to .5 were included in the heat map; data were row-normalized before plotting.

The gene expression patterns of the hypothalamic samples from 2 biological conditions can be characterized by a high-dimensional vector that consists of the read counts of all differentially expressed genes (DEGs). To visualize the similarity in the gene expression patterns, we conducted a principal component analysis (PCA) to transform the high-dimensional vectors into ones in a 3-dimensional space. In the PCA, the read counts in a vector were normalized by the size of the corresponding RNA-seq library. The 3 dimensions of the transformed space were chosen by the 3 principal components with the highest Eigenvalues. The Euclidean distance in the 3-dimensional space was used to measure the similarity of the gene expression patterns between the corresponding treatment group samples.

Hypothalamic genes that were significantly (*P* and *P*adj or q ≤ .05, at least 1.5 fold-change) dysregulated between any 2 dietary comparisons were uploaded to Panther (http://pantherdb.org) (34) for functional annotation clustering (35). Lists of various disease-associated mouse genes were generated using PubMed and cross-referenced with genes significantly altered between the diets as described before (14). Venn diagrams were created using the free online tool VENNY (36). The processed RNA-seq data are available online (37).

### Quantitative polymerase chain reaction

Following 18 to 26 weeks of chronic diet and GTT, mice were sacrificed by CO_2_ inhalation and decapitation. Whole brains were collected, snap-frozen in 2-methylbutane over dry ice, and stored at –80°C until later use. From each brain, the hypothalamus was dissected in half and homogenized in TRIzol Reagent (Thermo Fisher Scientific). Total hypothalamic RNA was prepared using a modified phenol-chloroform extraction protocol with the Qiagen RNeasy Plus Mini Kit. RNA concentration and purity were evaluated using a spectrophotometer (NanoDrop; Thermo Scientific). Reverse transcriptase (RT)-qPCR was performed with the SensiFAST SYBR No Rox One-Step Kit (Bioline) on the CFX Connect Real-Time PCR Detection System (Bio-Rad) using the following 2-step cycling protocol: 45°C for 10 minutes, 95°C for 2 minutes, followed by 40 cycles at 95°C for 5 seconds, 20 seconds at primer’s ideal annealing temperature, and melt curve analysis at 65°C and 95°C for 5 seconds. Each reaction was run in triplicate using 10 ng of RNA per reaction well.

Oligonucleotide primer sequences (Table 4) were custom designed and synthesized by Integrated DNA Technologies or ordered as predesigned assays. Primers were designed to meet several criteria using NCBI Primer Blast. For selectivity, primers were optimized by testing against complementary DNA generated from whole-mouse hypothalamus using RT-PCR and gel electrophoresis. Only primers that gave single-band amplicons in the presence of RT and that matched the base length of the predicted target were chosen for further annealing temperature optimization and efficiency checks via qPCR. A temperature gradient from 54°C to 62°C was used to determine optimal annealing temperature for each primer. Efficiency curves were generated over a 4- to 6-point template concentration range (100 ng-1 pg). Selected primers were optimized to yield 90% to 110% efficiency. No-template controls were run to rule out extraneous nucleic acid contamination and primer dimer formation. Negative RT controls were used to rule out any potential genomic DNA contamination present in the RNA preparation.

**Table 4.** Reverse transcriptase-quantitative polymerase chain reaction primers used in study

Primer	Accession No.	Sequence Forward 5’-3’ Reverse 5’-3’	T_m_°C, Forward/ Reverse	Product Length, bp	Primer Efficiency, %	Exon Target, Forward/ Reverse
*Oxt*	NM_011025.4	TTGGCTTACTGGCTCTGACCTC GGGAGACACTTGCGCATATCCAG	62.0/63.4	97	101.8	1/2
*Actb*	NM_007393 (1)	GACTCATCGTACTCCTGCTTG GATTACTGCTCTGGCTCCTAG	61.9/61.3	147	101.1	5/6

Abbreviations: *Actb,* β-actin; bp, base pair; *Oxt*, oxytocin; T_m_, melting temperature.

Samples were excluded from analysis if they had low amplitude peaks, 2 or more amplicons generated on melt curves, individual Cq values greater than 30, or having more than 1.0 SD from mean Cq of technical replicates. The fold-change gene expression of *Oxt* relative to *Actb* (β-actin) was determined using the Pfaffl method and biological replicates from CO as the control group (38). A total of 18 hypothalamus RNA samples from 18 mice were analyzed.

### Immunohistochemistry

At sacrifice, mice were anesthetized with isoflurane and subjected to cardiac puncture for collection of blood. They were immediately perfused transcardially with ice-cold 0.01 M phosphate buffer (PB) plus 0.9 g% NaCl (phosphate-buffered saline, PBS) followed by 4% paraformaldehyde (PFA) in phosphate buffer (pH 7.4). Brains were dissected, sucrose-infiltrated (30% sucrose), until brains were submerged (about 24 hours). Brains were cryosectioned into 30-μm sections and mounted onto gelatin-subbed microscope slides. Sections were washed free of PFA and subjected to a permeabilization/blocking step using 4% normal donkey serum, 1% bovine serum albumin, and 0.3% Triton-X in PBS. Sections were then incubated with 1 of 2 primary antibodies: mouse monoclonal anti-oxytocin neurophysin (PS38, 1:100; gift of Dr H. Gainer, National Institutes of Health) (39) for 48 hours at 4°C. This antibody has been shown to reliably detect levels of OXT (40). Sections were then washed in PBS 3 times and incubated with a secondary antibody (donkey antimouse Alexa 594, 1:1000) (41) at RT for 1.5 hours. After additional washes at 4°C, slides were cover-slipped using vectashield containing 4′,6-diamidino-2-phenylindole (DAPI; Vector Labs). OXT immunoreactivity of hypothalamic sections was analyzed under an Axiophot fluorescence microscope (Zeiss) and images acquired as fluorescent micrographs using a SPOT-II digital camera (Diagnostic Instruments). A mouse brain atlas (42) was referenced to ensure neuroanatomical comparison of equivalent brain regions. Seven experiments were performed using 3 sections from SON and PVN in each of 28 mice (n = 4-6/group).

### Oxytocin immunoassay

Blood was collected by cardiac puncture and the plasma was separated at 2000 × *g* for 20 minutes using a refrigerated centrifuge at 4°C. Samples underwent solid-phase extraction using 200 mg C18 Sep-Pak columns (Waters) using a modification of a published protocol (43). Columns were equilibrated with 3 mL of acetonitrile, then washed twice with 3 mL of 0.1% trifluoroacetic acid (TFA). Plasma (100 µL) was mixed with 4× volume of 0.1% TFA, centrifuged at 17 000 *g* for 15 minutes at 4°C, and the acidified and clarified plasma was then applied to the column. The columns were washed once with 15 mL of 0.1% TFA; the flow-through fraction was discarded. Analyte was collected by elution with 3 mL of 60% acetonitrile and completely dried under vacuum evaporation. Plasma samples were subjected to an enzyme-linked immunosorbent assay (ELISA) as indicated by manufacturer (Enzo Life Sciences) (44).

The assay is highly specific for OXT and does not detect *Avp*. Cross-reactivity of the kit is reported as mesotocin (7%), OXT (100%), Arg8-Vasotocin (7.5%), and less than 0.02% for other related molecules. Specifications of this kit are 15 pg/ml (or 1.5 pg/well) for limits of detection and 9% and 7% for intra-assay and interassay variability, respectively. The lyophilized sample was reconstituted in 0.12 mL of assay buffer provided in the kit and diluted 1:4 before processing. Briefly, standards and samples (100 μl) were loaded in duplicate onto a 96-well ELISA plate and processed as directed by kit specifications. Optical density (OD) was read at 405 nm; absorbance values were manually corrected using average OD values obtained from the control “blank” wells. OXT concentration values were determined according to a 4-parameter logistic fit standard curve. Values were reported as pictogram (pg) per mL. Extraction efficiency (recovery > 80%) was determined by spiking plasma with the 200 pg/ml OXT standard and comparing to standard plasma. Values obtained for control mouse subjects were similar to those reported previously (45). A total of 29 samples were evaluated for plasma OXT.

### Nongenetic statistical analysis

Data are presented as mean ± standard error of the mean. Hypothesis testing was conducted using statistical analysis (GraphPad Prism 6.0) using the following tests: repeated-measures two-way for effects of diet and time on body weight gain and food consumption, and one-way ANOVA for effects of diet on GTT, ELISA, and qPCR were followed by Tukey, Fisher least significant difference, and Bonferroni post hoc tests for multiple comparisons. For simple planned comparisons we used 1-tailed, unpaired Student t test. Statistical significance was accepted at an α level .05. Linear regression analysis was performed between body weight, glucose tolerance, and messenger RNA (mRNA) and plasma levels of OXT. The following cutoffs were used to determine significance: Pearson coefficient r greater than 0.5 with *P* less than or equal to .05 and R^2^ greater than 0.5. Sample sizes were calculated using power analysis at a desired statistical power of .8 and α level of .05 (www.stat.ubc.ca and masc.org.au\stats\PowerCalculator).

## Results

### Dietary oils have differential effects on hypothalamic gene expression

Transcriptomic analysis (RNA-seq) was performed on hypothalami obtained from adult male C57BL6/N mice used in a previous study (cohort 1) in which we reported that an HFD made with conventional SO (SO + CO) induces more obesity than isocaloric diets made with either CO or a genetically modified low-LA soybean oil, PL (PL + CO) (13) (Fig. 1A). As described previously (14), these diets were designed to have a level of total fat (40% kcal) and LA (10% kcal) comparable to the current American diet. Coconut oil was used as the HFD control because it is composed nearly exclusively of saturated fats and contains no endogenous LA; a small amount of SO had to be added because LA is an essential fatty acid (see Tables 1 and 2).

**Figure 2.**
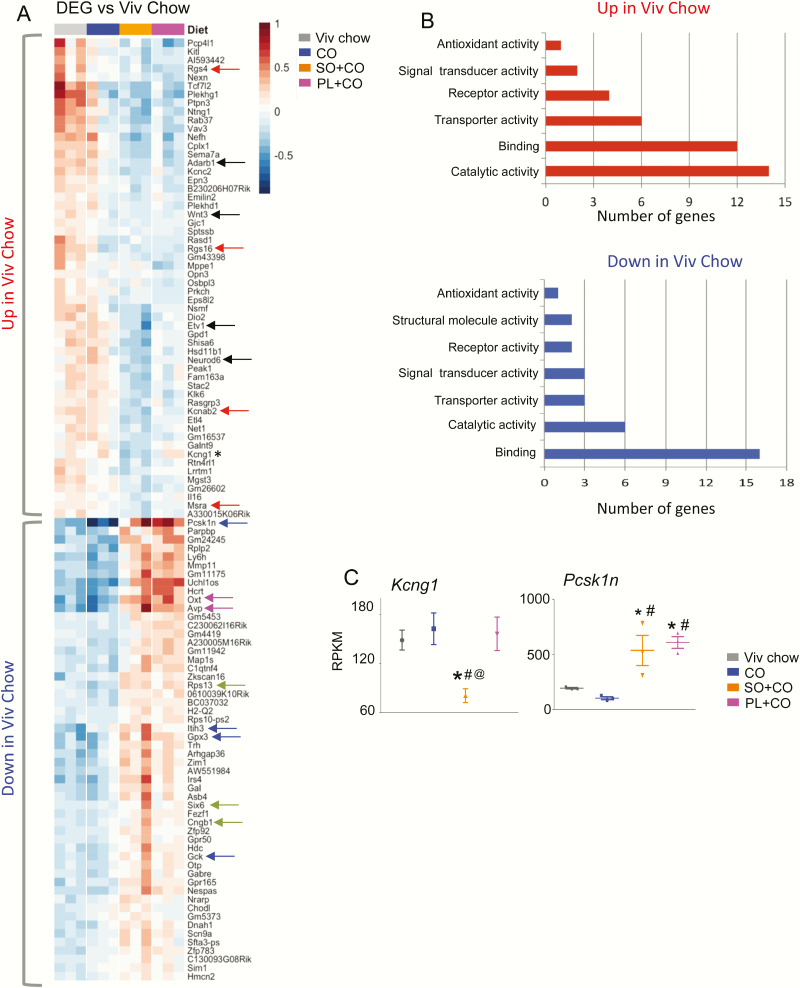
Soybean oil affects the hypothalamic transcriptome. A, Heat map of differential hypothalamic gene expression (RNA sequencing [RNA-seq]) between mice fed vivarium (Viv) chow, CO, SO + CO, and PL + CO diets for 24 weeks. Color bar indicates level of gene expression relative to the whole data set. Top, genes upregulated in Viv chow vs the 3 high-fat diets. Bottom, genes downregulated in Viv chow. Arrows, genes mentioned in text. Asterisk, *Kcng1,* the only gene significantly different between SO + CO and PL + CO. B, Gene ontology showing categories of molecular function affected by diet. C, Absolute expression levels from RNA-seq of indicated diets. DEG, differentially expressed genes; RPKM, reads per kilobase per million. Statistically different from ^#^CO, *Viv chow, ^@^PL + CO. CO diets enriched in conventional soybean oil (SO + CO), genetically modified Plenish (PL + CO), and stigmasterol (ST + CO).

PCA showed that both SO diets significantly alter gene expression in the hypothalamus compared to the CO diet, which was very similar to the low-fat Viv chow control diet (Fig. 1B). In fact, a heat map of DEGs significantly (log2 fold-change ≥ .5 and *P*adj ≤ .5) dysregulated in at least 1 of the 3 HFDs vs Viv chow revealed a nearly identical transcriptome in the CO diet and Viv chow: only 5 genes were significantly different (*Col11a1, Rgs16, Gm43398, Atp2a1*, and *Ppp1r3a*) (see [37] for absolute levels of these genes). In addition, the 2 SO diets (SO + CO and PL + CO) have a similar pattern of gene expression, which is roughly the inverse of that observed in Viv chow–fed and CO-fed mice (Fig. 2A). In fact, expression of only one gene was significantly different between SO + CO and PL + CO: *Kcng1* (Fig. 2A, asterisk and Fig. 2C) as well as a putative gene *1700018M17Rik* (not shown)*. Kcng1* is a potassium voltage-gated channel modifier (subfamily G member 1) that belongs to a family of proteins that regulate neurotransmitter release, among other physiological functions: its expression was downregulated approximately 2-fold in the conventional SO compared to the Viv chow but not changed by either CO or PL.

The top half of the heat map contains the genes that have a higher level of expression in Viv chow–fed mice compared to at least one of the HFD groups (“Up in Viv Chow”), whereas the bottom half contains the genes that have lower levels of expression in Viv chow–fed hypothalami (“Down in Viv Chow”). Gene ontology (GO) analysis of the molecular functions associated with the dysregulated genes revealed that the majority of the genes in “Up in Viv Chow” (down in SO + CO and PL + CO) are categorized as “binding” and “catalytic activity” genes (Fig. 2B top). The binding category contains genes that code for nucleic acid or DNA binding proteins such as transcription factors *Etv1* and *Neurod6* and RNA adenosine deaminase *Adarb1* (Fig. 2A, solid black arrows). The proto-oncogene *Wnt3*, which signals to the transcription factor β-catenin and the neuropeptide genes *Oxt* and *Avp* (Fig. 2A, pink arrows), are also included in the binding category. The catalytic activity genes suppressed by the SO diets (“Up in Viv Chow”) include G-protein modulators (*Rgs4* and *Rgs16)*, a voltage-gated potassium channel (*Kcnab2*), and a mitochondria reductase (*Msra)* (Fig. 2A, red arrows). The genes “Down in Viv Chow” (up in SO + CO and PL + CO) were also primarily in the “binding” and “catalytic activity” categories (Fig. 2B bottom). The binding category includes *Rps13, Six6*, and *Cngb1* (Fig. 2A, green arrows), whereas the catalytic activity category includes protease inhibitors (*Itih3, Pcsk1n)*, glucokinase (*Gck)*, and a peroxidase (*Gpx3)* (Fig. 2A, blue arrows, and Fig. 2C). (Genes included in each category shown in [37]).

**Figure 3.**
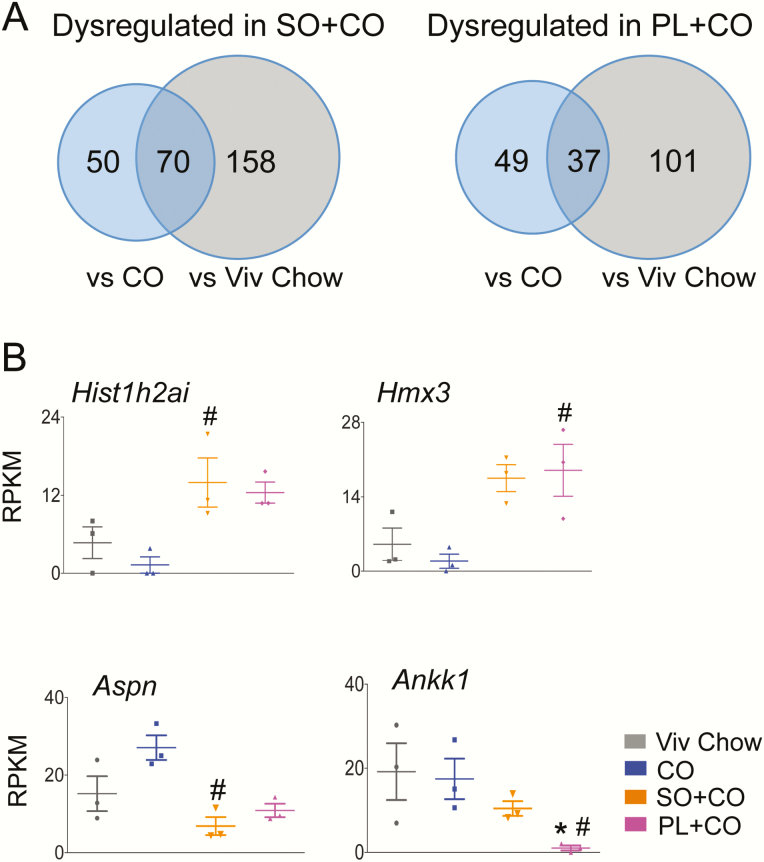
Comparison of differentially expressed hypothalamic genes in different diet groups. A, Venn diagram of number of genes dysregulated in left, SO + CO, and right, PL + CO vs CO and Viv chow diets (≥1.5-fold, *P* and *P*adj ≤.05). B, Absolute expression levels from RNA sequencing (RNA-seq) of genes that are most dysregulated between SO + CO, PL + CO, or both soybean oil diets vs CO. Statistically different from ^#^CO, *Viv chow. CO diets enriched in conventional soybean oil (SO + CO), genetically modified Plenish (PL + CO), and stigmasterol (ST + CO).

When compared to the Viv chow–fed mice, there were 228 hypothalamic genes that were significantly dysregulated in SO + CO and 138 genes in PL + CO–fed mice (Fig. 3A) (37). There was a smaller number of genes dysregulated in the 2 SO diets when compared to the isocaloric CO diet: 120 genes (87 upregulated and 33 downregulated) in SO + CO and 86 genes (82 upregulated and 4 downregulated) in PL + CO (Fig. 3A) (37). *Hist1h2ai,* a gene involved in nucleosome assembly (46), was increased the most (~11-fold) in SO + CO, and *Hmx3,* a transcription factor associated with hypothalamic development and neuroendocrine function (47,48), was increased the most in PL + CO (~10-fold) (Fig. 3B top). For both SO diets, the predicted gene *Gm44367* was the most upregulated (18-fold, not shown). *Aspn,* which is involved in regulating inflammatory responses (49), and *Ankk1* were the most downregulated genes in SO + CO (~3-fold) and PL + CO (~21-fold) vs CO, respectively (Fig. 3B, bottom). (The putative gene *1700018M17Rik* was also highly downregulated, not shown). Interestingly, *Ankk1* alters dopamine D2 receptor signaling; genetic variants with it have been associated with obesity (50), neuropsychiatric disorders, impulse control disorder and exercise reinforcement (51,52).

### Hypothalamic genes dysregulated by soybean oil are linked to neuroendocrine, neurochemical, signaling, and gene regulation pathways

**Figure 4.**
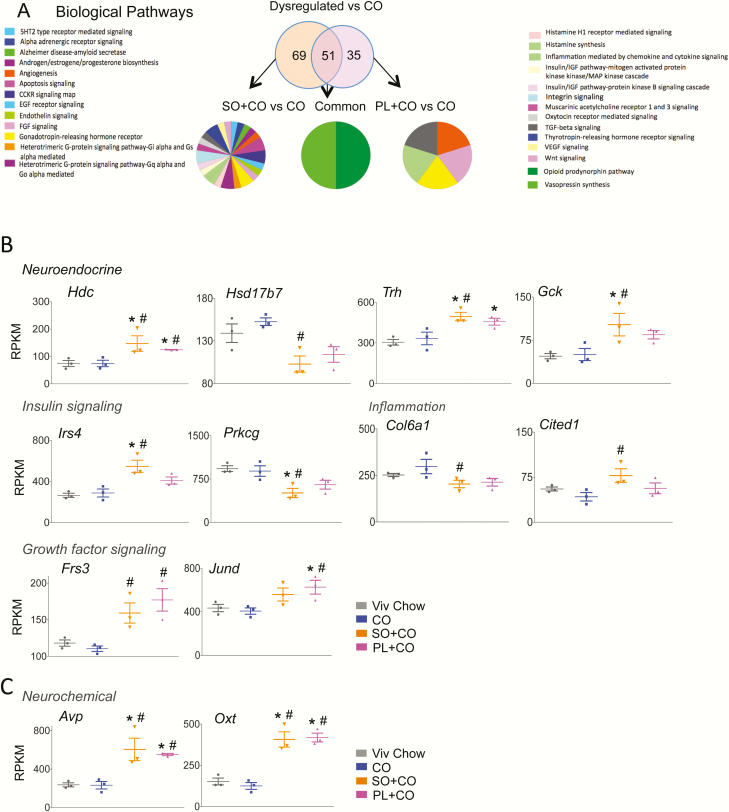
Biological pathways associated with hypothalamic genes dysregulated by soybean oil (SO)-rich high-fat diets. A, Venn diagram showing number of genes dysregulated (≥ 1.5-fold, *P* and *P*adj ≤ .05) either exclusively or commonly by SO + CO and PL + CO vs CO and gene ontology for biological pathways altered in the comparisons shown. B, Absolute expression levels from RNA sequencing of dysregulated genes (≥ 1.5 fold, *P* and *P*adj ≤ .05) in the most prominent pathways. Significantly different from ^#^CO, *Viv chow. CO diets enriched in conventional soybean oil (SO + CO), genetically modified Plenish (PL + CO), and stigmasterol (ST + CO).

GO analysis was performed on the genes dysregulated vs CO that were common in both SO diets (51 genes), as well as the genes uniquely dysregulated by SO + CO (69 genes) and PL + CO (35 genes) (Fig. 4A) (37). Of the 69 genes dysregulated in SO + CO vs CO, only 31 could be matched to 1 of 27 biological pathways, including neurochemical and neuroendocrine pathways, inflammatory processes, and insulin signaling (Fig. 4A). The neurochemical and neuroendocrine pathways represented here include cholecystokinin signaling (*Hdc)*, thyrotropin-releasing hormone receptor (*Trh),* gonadotropin-releasing hormone receptor *(Gck)*, and sex steroid hormone biosynthesis (*Hsd17b7)* (Fig. 4B). Dysregulated genes involved in inflammation include *Col6a1*, which signals via chemokines or cytokines, and *Cited1*, a *Cbp/p300* interacting transactivator that signals through transforming growth factor β (TGF-β) (Fig.4B). Dysregulated genes involved in insulin signaling include *Irs4* (insulin receptor substrate 4) and *Prkcg* (protein kinase C γ) (Fig. 4B). In addition to insulin signaling, *Prkcg* is involved in acetylcholinergic, adrenergic, oxytocin receptor-mediated, thyrotropin-releasing hormone, and Wnt signaling and has a wide distribution and range of function in the brain (53-55). The expression and/or function in the brain has also been documented for *Hdc* (56, 57), *Trh* (58)*, Cited1* (59, 60), *Gck* (61), and *Irs4* (62).

In the PL + CO vs CO comparison, 2 of the 35 dysregulated genes appeared in multiple pathways: *Frs3*, which affects angiogenesis and fibroblast growth factor signaling, and *Jund,* which modulates gonadotropin-releasing hormone receptor, inflammation, and TGF-β mediation (Fig. 4B). Only 2 of the 51 commonly dysregulated genes between SO + CO vs CO and PL + CO vs CO were associated with biological pathways in the GO analysis: *Avp* and oxytocin (*Oxt)* (both increased ~3-fold compared to CO) (Fig. 4C) are associated with opioid prodynorphin and *Avp* synthesis. Interestingly, hypothalamic expression of *Jund* is upregulated following physiological activation of *Avp* and oxytocinergic MNCs of the SON (63) (Fig. 4B). It is worth noting that all the SO-dysregulated genes highlighted in Fig. 4 trended in the same direction in the high-LA (SO + CO) and low-LA (PL + CO) diets, even if the expression in one of the diets did not quite reach significance.

### Hypothalamic genes dysregulated by dietary fat are linked to several types of molecular functions, including transcriptional regulation

GO analysis with respect to molecular function revealed additional genes of interest dysregulated by the SO diets (37). The SO + CO diet resulted in an upregulation of *Gal*, *Gabre, Gpr50*, and *Mc3r*, and downregulation of *Sema7a* and *Shisa6.* In the PL + CO vs CO comparison, only one gene was dysregulated: *Crlf2* (up). The transcriptional regulators that were dysregulated in SO + CO vs CO include *Neurod6* (down), *Etv1* (down), and *Six6* (up). *Neurod6* is known to play a role in neurogenesis in the hypothalamus (64). Hypothalamic expression of *Etv1* evokes an increase in water-intake behavior (65). *Six6* is required for neuroendocrine development, specifically that of the suprachiasmatic nucleus, the expression of which is regulated in a sex-specific and circadian manner (66,67).

### Hypothalamic genes dysregulated by soybean oil diet are linked to metabolic diseases and inflammation

To reveal additional links between the SO diets and metabolic disease, we compared PubMed-generated lists of genes associated with diabetes, lipid metabolism, obesity (grouped as metabolic disease–related), and inflammation to the hypothalamic DEGs. Eighteen disease-associated genes were identified across the 4 categories for SO + CO vs CO, of which 11 were uniquely dysregulated by SO + CO: *Hsd11b1, Gck, Hdc, Ghsr, Hsd17b7, Gal, Dio2, Mc3r, Gpx3, Nr4a2*, and *Sema7a* (presented in order of decreasing fold-change) (Table 5). In contrast, there were 9 disease-associated genes that were identified for PL + CO vs CO, with *Mt1* and *Crlf2* being the only genes uniquely dysregulated by PL + CO (Table 5). The genes commonly dysregulated in both SO diets were *Hcrt, Oxt, Avp, Cartpt, Mmp11*, and *Abhd8* (Table 5). Interestingly, when compared to Viv chow, both SO + CO and PL + CO diets caused dysregulation of 8 to 22 genes associated with metabolic disease (37), whereas the CO diet yielded only 2 disease-related genes (*Atp2a1* and *Rgs16*), both of which are linked to inflammation. This suggests that the medium-chain fatty acids in CO not only have less of an impact on hypothalamic gene expression in general, but also in terms of gene expression related to metabolic and inflammatory diseases, than the 2 SO diets (Table 5).

**Table 5.** Dysregulated hypothalamic genes related to metabolic disease and inflammation in male mice (*P* ≤ .05, q ≤ 0.05, FC ≥ 1.5)

	Metabolic Disease			
Diet	Diabetes	Lipid Metabolism	Obesity	Inflammation
SO + CO vs CO	*Oxt, Mmp11, Hcrt, Avp, Hsd11b1, Gck, Hdc, Cartpt*	*Hsd11b1, Ghsr, Gck, Abhd8, Hsd17b7*	*Mc3r, Hsd11b1, Hcrt, Ghsr, Gal, Gck, Cartpt, Gpx3, Dio2*	*C1qtnf4, Oxt, Hsd11b1, Ghsr, Cartpt, Nr4a2, Dio2, Sema7a*
PL + CO vs CO	*Hcrt, Mmp11, Oxt, Avp, Cartpt, Mt1*	*Abhd8*	*Hcrt, Cartpt, Mt1*	*C1qtnf4, Oxt, Crlf2, Cartpt, Mt1*
CO vs Viv	0	0	0	*Atp2a1, Rgs16*

Genes are presented in order of decreasing fold-change.

Abbreviations: CO, coconut oil; PL, Plenish; SO, soybean oil; Viv, vivarium chow.

### Hypothalamic genes dysregulated by soybean oil diet are linked to neurological diseases

**Figure 5.**
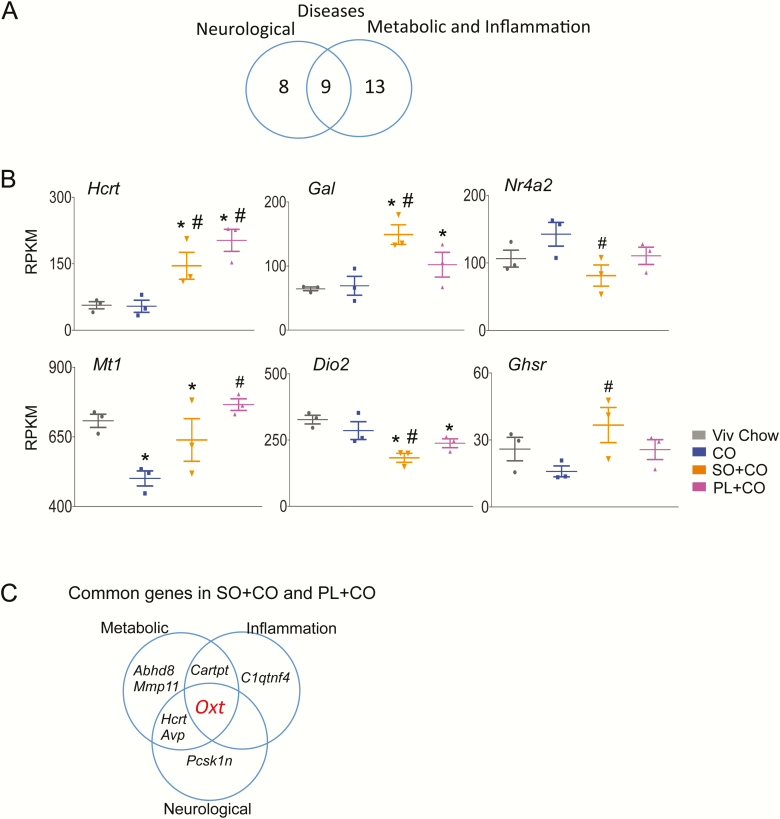
*Oxt* is the only gene dysregulated in both soybean oil diets (vs CO and Viv chow) that is associated with neurological, metabolic, and inflammation disease categories. A, Venn diagram showing overlap in hypothalamic genes from RNA sequencing (RNA-seq) related to neurological diseases vs metabolic diseases and inflammation dysregulated in SO + CO and PL + CO vs CO (Tables 5 and 6). B, Absolute expression levels from RNA-seq data of 6 of the 9 common genes. Significantly different from ^#^CO, *Viv chow. C, Venn diagram showing overlap in genes related to metabolic diseases (obesity, diabetes, and lipid metabolism), inflammation, and neurological diseases that are dysregulated in the hypothalamus of both soybean oil diets vs CO diet. Oxytocin (*Oxt*, red font) is the only gene common to all 3 disease categories and is significantly different both from CO and Viv chow. CO diets enriched in conventional soybean oil (SO + CO), genetically modified Plenish (PL + CO), and stigmasterol (ST + CO).

A similar analysis was performed to identify hypothalamic DEGs related to neurological diseases, using *Alzheimer disease, anxiety, autism, depression, pain, Parkinson disease*, and *schizophrenia* as search terms in PubMed. As with inflammation and metabolic diseases, more genes in the SO + CO vs CO comparison were identified in one or more categories than the PL + CO vs CO comparison. No genes associated with neurological diseases were identified among the CO vs Viv chow DEGs (Table 6). A Venn analysis of dysregulated genes in SO + CO and PL + CO vs CO that are associated with metabolic diseases plus inflammation categories (Table 5) and with neurological disorders (Table 6) reveal 9 common genes (Fig. 5A). Those genes include *Hcrt* (metabolic disease, anxiety, depression, and pain)*, Gal* (metabolic disease, Alzheimer, anxiety, depression, and pain), *Nr4a2* (inflammation, Parkinson disease, and schizophrenia)*, Mt1* (metabolic disease, inflammation, and depression), *Dio2* (metabolic disease, inflammation, anxiety, and depression), *Ghsr* (metabolic disease, inflammation, pain, and Parkinson disease) (Fig. 5B) and *Hdc* (metabolic disease and anxiety), *Avp* (metabolic disease, anxiety, and pain), *Oxt* (metabolic disease, inflammation, anxiety, autism, depression, and pain) (Fig. 4B,C). Importantly, *Oxt* was the only gene dysregulated in *both* SO diets vs *both* CO and Viv chow and also associated with all 3 disease categories: metabolic disease, inflammation, and neurological disorders (Fig. 5C).

**Table 6.** Dysregulated hypothalamic genes related to neurological disease in male mice (*P* ≤ .05, q ≤ 0.05, FC ≥ 1.5)

Diet	Alzheimer’s	Anxiety	Autism	Depression	Pain	Parkinson’s	Schizophrenia
SO + CO vs CO	*Pcsk1n, Gal, Irs4,Ngb, Nrn1*	*Oxt, Hcrt, Avp, Gal, Hdc,Dio2, Baiap3*	*Oxt*	*Oxt, Hcrt, Avp, Gal, Dio2*	*Oxt, Hcrt, Ghsr Gal, Prkcg*	*Ghsr, Nr4a2*	*Nr4a2*
PL + CO vs CO	*Pcsk1n*	*Hcrt, Oxt, Avp*	*Oxt, Nrxn2*	*Hcrt, Oxt, Avp, Mt1*	*Hcrt, Oxt*	*0*	*Nrxn2*
CO vs Viv	0	0	0	0	0	0	0

Genes are presented in order of decreasing fold-change.

Abbreviations: CO, coconut oil; PL, Plenish; SO, soybean oil; Viv, vivarium chow.

### Metabolic phenotype induced by soybean oil is not due to stigmasterol

**Figure 6.**
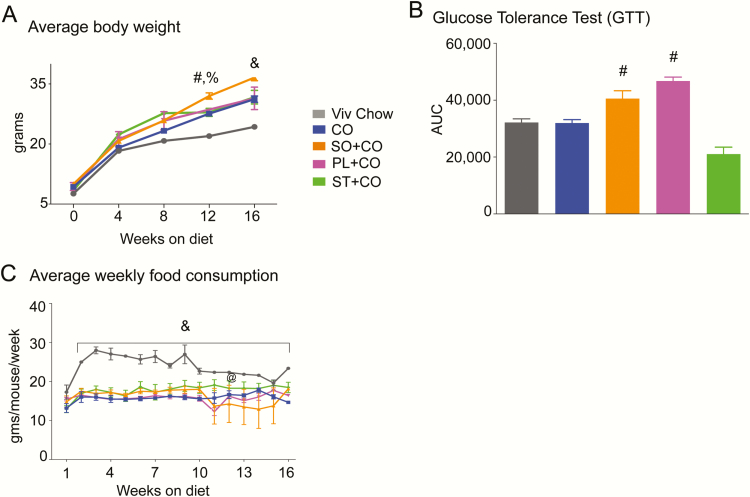
Obesogenic and diabetogenic effects of soybean oil (SO) diets with high or low linoleic acid (LA) is not mimicked by stigmasterol. A, Average weekly body weights of male C57BL/6N mice started on the indicated diets at weaning (N = 6-12/diet) as in Fig. 1A. All diets were isocaloric with 40 kcal% total fat except Viv chow, which had 13.4 kcal% fat. B, Glycemia (mg/dL) measured during a glucose tolerance test (GTT) depicted as area under the curve after 16 weeks on diet (N = 6–12/diet). C, Average weekly food consumption of mice on various diets measured on a per-cage basis and normalized to the number of mice per cage. Food was changed and measured twice weekly; values were combined to generate the weekly average. Consumption of Viv chow was highest because this diet has the fewest calories per gram (N = 6-12 mice [3-4 cages]/diet). ^@^Statistical difference between PL + CO and ST + CO at 12 weeks on diet. Statistically different from ^#^CO, ^%^ST + CO, ^&^all others using one-way analysis of variance followed by Tukey post hoc analysis for B or two-way ANOVA followed by Tukey post-hoc analysis for A and C.

Because the RNA-seq analysis of the hypothalamus of mice fed both the conventional, high-LA (SO) and genetically modified, low-LA (PL) SOs revealed similar effects on the expression of *Oxt* (and many other genes), we designed a second cohort of mice to determine whether another component of the oils—ST—might play a role in the altered *Oxt* expression and obesity and diabetes. Mice in cohort 2 (Fig. 1A) were fed Viv chow, CO, SO + CO, PL + CO and ST + CO (CO diet supplemented with 0.1 g ST per 1.1 kg diet to match the level in the 2 SO diets, Table 1). Consistent with our previous studies (13, 14), we observed that all 3 HFDs in cohort 2 mice fed CO, SO + CO, or PL + CO experienced a greater gain in body weight than the Viv chow–fed mice after 16 weeks on the diet. A two-way ANOVA showed main effects of duration on diet (F_4,179_ = 824, *P* < .001) and diet (F_3,179_ = 91 *P* < .001). Post hoc group comparisons indicated that the SO + CO group gained the most weight (36.6 vs 24.3 g for Viv chow) (Fig. 6A). However, there was no significant difference in body weight in PL + CO mice compared to the CO mice in cohort 2 after 16 weeks on the diet (31.4 and 31.2 g, respectively) (Fig. 6A), as we showed previously (13). This discrepancy could be because the mice in cohort 2 were on the diets for only 16 weeks compared to 24 weeks in cohort 1: The difference in weight between PL + CO and CO mice in that study did not gain significance until 12 weeks on the diet (13). Another possible explanation is that the mice in the 24-week study (cohort 1) were housed in a specific pathogen-free animal facility, whereas mice for the 16-week study (cohort 2) were housed in a conventional vivarium, potentially implicating the microbiome. Both SO diets (SO + CO and PL + CO) made the mice more glucose intolerant compared to the CO and Viv chow diets (Fig. 6B), also consistent with our previous results (13).

Interestingly, we found that mice fed the ST + CO diet gained less weight compared to SO + CO mice but the same amount of weight (31.7 g) as those on the CO and PL + CO diets. The ST + CO-fed mice also showed no signs of glucose intolerance in the GTT and were trending toward even less glucose tolerance than the CO and Viv chow diets (Fig. 6A and 6B). A one-way ANOVA on area under the curve for different diets showed a main effect of diet (F_4,28_ = 7.130, *P* < .001). The reduced weight gain was not due to lower food consumption by the ST + CO mice because the mice on all the HFDs displayed similar food intake (Fig. 6C). Only the Viv Chow mice showed greater food consumption relative to HFD diets (F_15,60_ = 2.2 *P* < .01 for time on diet and F_2.2,33_ = 69, *P* < .001 for diet). Taken together, we conclude that the obesogenic and diabetogenic component of conventional SO is a compound other than ST. As noted previously, LA could be contributing to obesity but not to glucose intolerance (13).

### Changes in oxytocinergic system due to dietary soybean oil are independent of stigmasterol

**Figure 7.**
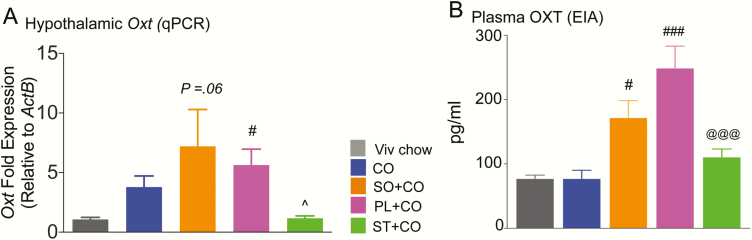
Dietary soybean oil (SO) dysregulates hypothalamic gene expression and plasma levels of oxytocin. C57BL/6N male mice fed Viv chow, CO, SO + CO, PL + CO, or ST + CO diets for 17 to 28 weeks. A, Transcript levels of *Oxt*, expressed relative to β-actin (*Actb)* measured in hypothalamic homogenates using reverse transcriptase-quantitative polymerase chain reaction and expressed relative to levels in CO using the Pfaffl method (N = 3-6/diet). B, Peripheral oxytocin peptide levels measured via EIA, (N = 3-10/diet). Statistically different from: ^#^CO; ^@@@^PL + CO; ^^^SO + CO and PL + CO (single symbols, *P* < .05; triple symbols *P* < .001) using 1-way analysis of variance (ANOVA) followed by Tukey post hoc analysis for A and one-way ANOVA followed by Bonferroni post hoc analysis for B.

To determine whether ST affected *Oxt* expression, we performed RT-qPCR on RNA isolated from the hypothalamus of male mice fed the various diets (Fig. 7A). A one-way ANOVA showed a main effect of diet (F_3,14_ = 4.82 *P* < .05, N = 3-6/diet). Post hoc analysis revealed lower *Oxt* expression in the ST-enriched diet (ST + CO) compared to either of the 2 SO diets (*P *< .*05*). In contrast, Pfaffl ratios for *Oxt* transcript levels were significantly upregulated in PL + CO (*P *< .05) but were just below the level of significance in SO + CO (*P *= .06) relative to CO, consistent with the RNA-seq results.

Blood drawn at the time of death was used to measure plasma levels of oxytocin using an ELISA (Fig. 7B). One-way ANOVA showed main effects of diet (F_4,24_ = 12.84, *P* < .001, N = 3-10/diet). Post hoc group comparisons revealed that plasma OXT in SO + CO (*P* < .05) and PL + CO diets (*P* < .001) was significantly elevated compared to CO, consistent with the notion that these changes are likely due to a component of SO. That component, however, was not ST because the ST + CO-fed mice had similar levels to those in the CO diet group but significantly lower plasma OXT compared to the PL + CO group (*P* < .001).

An analysis was performed to correlate gene expression and plasma levels of OXT with body weight and GTT in each of the dietary groups (37). The only correlation that we found to be significant was the *Oxt* mRNA vs GTT (r = 0.9, *P* = .04). The plasma OXT vs GTT was trending toward significance (r = 0.8, *P* = .06). In contrast, there were no significant correlations with body weight. Similarly, a heat map of individual body weights of mice used for RNA-seq analysis did not show the same pattern as the gene expression profiles in Fig. 2A (37).

### Central oxytocin levels are dysregulated by components of soybean oil

**Figure 8.**
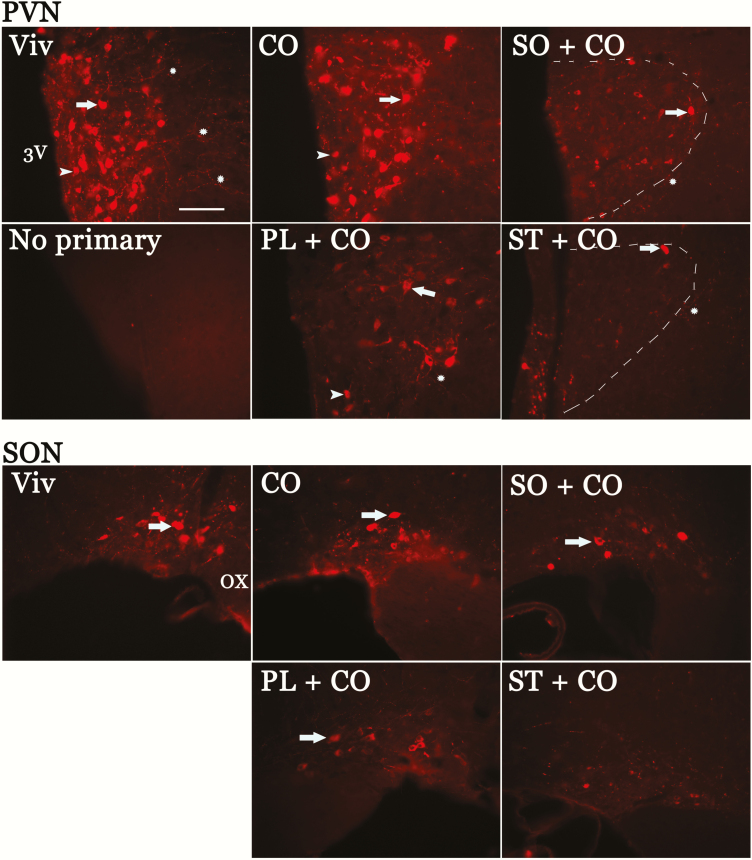
Dietary soybean oil decreases oxytocin immunoreactivity in the magnocellular neuroendocrine nuclei of the hypothalamus. Oxytocin-neurophysin immunoreactivity in micrographs of hypothalamic tissue sections from perfused brains of male mice fed Viv chow (Viv), CO, SO + CO, PL + CO, or ST + CO diets for 17 to 28 weeks. Reduced immunofluorescence was observed in SO + CO, PL + CO, and ST + CO compared to CO and Viv chow in the large magnocellular neuroendocrine of the SON and PVN and smaller parvocellular neurons of the PVN. 3V, third ventricle; arrow, magnocellular neuroendocrine cells; arrowhead, parvocellular neurons; asterisk, immunolabeled axonal projections; dashed line, PVN boundary in SO + CO and ST + CO; OX, optic chiasm; PVN, paraventricular nucleus; SON, supraoptic nucleus. Calibration bar = 100 μm.

To determine the abundance of OXT protein in the brain, hypothalamic tissue sections were incubated with an antibody that recognizes OXT-neurophysin, a carrier protein for OXT: Cell bodies, dendrites, and axonal projections of OXT-positive neurons were observed in the SON and PVN of the hypothalamus (Fig. 8). Within the PVN and SON both MNCs (with cell body size > 25 μm) and PVN parvocellular neurons (cell bodies < 25 μm) are intensely stained in Viv chow– and CO-fed groups. In contrast, the density of those networks and the intensity of the signal was markedly decreased in the other HFDs: SO + CO, PL + CO, and ST + CO. Seven IHC experiments were performed using 4 to 6 mice per group with similar results.

## Discussion

The consumption of SO, one of the most ubiquitous components of the American diet, has increased dramatically over the last several decades and parallels the increase in prevalence both of obesity and diabetes in humans (10, 18). We and others have shown previously that HFDs enriched in SO induce both obesity and diabetes in rodent systems (13-15). In the present study, we show that diets enriched in either conventional SO high in LA (SO + CO) or a genetically modified SO low in LA (and high in oleic acid), Plenish (PL + CO), made male mice more glucose intolerant compared to the control HFD, CO. In addition, the SO + CO diet caused more weight gain over the 16-week study than the other HFDs. Importantly, both SO-rich diets produced a considerable dysregulation of gene expression in the hypothalamus of male mice, the most notable of which is the gene coding for *Oxt*. We also observed elevated levels of circulating OXT peptide in the plasma of SO-fed mice. An isocaloric CO diet did not affect the expression of *Oxt* either in the brain or the circulation, and had a minimal effect on hypothalamic gene expression compared to the low-fat control diet. Furthermore, the level of neither LA nor ST in the diets had a significant effect on hypothalamic *Oxt* mRNA or plasma OXT. All told, our results demonstrate that different dietary oils can have differential effects on hypothalamic gene expression and raise the possibility that the SO-rich American diet may be not only contributing to increased rates of metabolic disease but also affecting neurological function.

Chronic SO + CO and PL + CO diets produced similar hypothalamic transcriptomic profiles and dysregulated more genes than CO when compared to the low-fat control Viv Chow diet (Fig. 2A). Although this suggests that components other than LA may be responsible for the majority of the effects on the hypothalamus, some subtle differences were noted between the low-LA and high-LA diets. The pathways altered by SO + CO include neuroendocrine, inflammation, and insulin signaling, and those altered by PL + CO include growth factor signaling. Neurochemical signaling pathways for opioid prodynorphin and *Avp* synthesis were common to both because of 2 genes: *Oxt* and *Avp*.

### Impact of soybean oil diet on oxytocinergic system

Oxytocin signaling within the central nervous system and oxytocin hormone secretion into the periphery play important roles in regulating energy balance, body weight gain, and glucose homeostasis (2, 68, 69). Central administration of OXT reduces diet-induced obesity, and antagonism of central OXT receptors is obesogenic (70). In particular, *central* OXT within the PVN, an area critical for energy homeostasis and susceptible to the effects of HFD, can influence body weight regulation, as has been shown for OXT and other hypothalamic neuropeptide systems previously (71, 72). The role of peripheral OXT in regulating obesity and diabetes is more controversial because some studies report that circulating OXT can decrease body weight, fat mass and glucose intolerance (73, 74), whereas others find a positive association between plasma OXT and body mass index and glucose intolerance in humans (69, 72, 75). What is clear is that the metabolic functions of the oxytocinergic system are complex and dependent both on *central* and *peripheral* actions.

In the current study, HFDs enriched in conventional SO (SO + CO) and low-LA Plenish (PL + CO) had effects both on the peripheral and central components of the oxytocinergic system. Plasma OXT peptide levels were increased in the blood: ~250% (PL + CO) and ~200% (SO + CO) relative to both the high-fat CO and low-fat control (Viv chow) diets. Although *Oxt* transcript levels in the hypothalamus were also significantly increased in both SO diets, IHC experiments showed decreased OXT immunoreactivity in parvocellular cells and MNCs of the SON and PVN. This suggests a potential impact on central OXT release from MNC dendrites and synaptic neurotransmission as well as peripheral release of OXT from MNC axons that project to the posterior pituitary and release OXT into the systemic circulation (3, 4). Others have shown that a chronic HFD (composition unspecified) results in elevated *Oxt* mRNA in the murine hypothalamus but blunts the stimulated release of central OXT from PVN slices manipulated ex vivo (76), suggesting that the increased *Oxt* transcription could be a compensatory mechanism for suppressed OXT release.

Regardless of which aspect of OXT function—central or peripheral—is more relevant to the metabolic effects of SO, what is clear is that LA does not appear to play a role. Mice fed conventional SO, high in LA (~55%), or the genetically modified PL, low in LA (7.4%), displayed essentially identical effects on every aspect of oxytocin examined—elevated RNA levels in the hypothalamus, low protein immunoreactivity in SON and PVN, and high peptide levels in plasma. Although others have reported that PUFAs from various dietary sources (eg, safflower and sunflower seed oils that are also high in LA) induce obesity in rodents (16, 23, 24), they did not examine changes in *Oxt.* They did report, however, an increase in expression of other hypothalamic neuropeptide genes such as *POMC*, *GALP*, *MCH*, *preORX, Agrp*, and *Cart* (also known as *Cartpt)* as well as changes in proinflammatory genes *Akt*, *Ikk*, *IL6*, *Icam1*, and *Cx3cl1*. We did not observe a change of expression in any of these genes with either SO diet, with the exception of *Cartpt*, *Pmch* (precursor to melanocortin concentrating hormone, MCH) and *Akt1s1,* a substrate of AKT, reinforcing the notion that the oxytocin-related changes observed in the SO diets are likely due to some component other than LA.

Like LA, the addition of ST to the CO diet (ST + CO) did not increase the levels of *Oxt* RNA in the hypothalamus nor OXT peptide in the plasma. However, the mice fed the ST + CO diet did show light hypothalamic OXT immunoreactivity, as did the 2 SO diets, suggesting that ST could be affecting the synthesis and/or central release from the oxytocinergic neurons. Finally, it is worth noting that although we have examined only male mice in this study, the effects of SO diets on the female hypothalamus still needs to be addressed in future studies. We do not *a priori* expect sex differences produced by dietary SO on solely this basis because SO is free of phytoestrogens, such as isoflavanoids (77). However, because the hypothalamic oxytocinergic system shows sexual dimorphism, especially with regard to maternal behavior, there may be potential sex differences resulting from dietary SO.

### Correlation between oxytocin gene and peptide expression and body weight and glucose intolerance in soybean oil diets

An alternative hypothesis to the SO diets directly affecting the oxytocinergic system is that they exert effects on peripheral organs such as liver, adipose, and muscle, causing obesity and diabetes, and that it is those metabolic states that affect *Oxt* expression rather than the constituents of the diets, or their metabolites, *per se*. Correlation analysis between body weight gain and glucose intolerance vs hypothalamic *Oxt* mRNA and circulating OXT peptide shows that although the obese state per se is not significantly correlated with *Oxt* levels, the diabetic state is (as measured by glucose intolerance). Indeed, others have reported that both spontaneous and induced diabetes results in an increase in hypothalamic Oxt mRNA and an increase in hypothalamic OXT protein immunointensity (5). Arguing against the notion that it is the diabetic state and not the diets directly that alter the oxytocinergic system is the fact that the PL diet does not lead to another facet of diabetes, insulin resistance, whereas conventional SO does (13). Nonetheless, if some aspect of the metabolic state does, in fact, affect *Oxt* expression, then the question arises about the nature of the underlying cause(s) of the changes in expression of non-metabolic-associated genes observed in the SO + CO and PL + CO diets (Fig. 2A). Finally, it is worth noting that although oxytocin is well known to regulate body weight, in part, by influencing eating behavior and satiety (2, 72), a significant difference in the amount of food consumed between the different diets was not observed (Fig. 6C).

### Impact of soybean oil diet on other genes linked to metabolic disease

In addition to *Oxt*, there were several other genes related to metabolic function that were dysregulated by the SO diets. Most notable of those was the neuropeptide *Avp*, which, like OXT, is expressed primarily in the SON and PVN and associated with body weight regulation and diabetes (72, 78) and was upregulated in the hypothalamus of the SO + CO and PL + CO diets. Peripheral AVP has been recognized as a risk factor for type 2 diabetes (79), but the role of hypothalamic AVP in diabetes is still being explored (78).

Other genes commonly dysregulated in both SO diets linked to metabolic disorders include *Cartpt, Hcrt, Mmp11*, and *Abhd8* (all upregulated) (Fig. 5C). *Cartpt* (cocaine- and amphetamine-regulated transcript) gives rise to bioactive neuropeptides that are associated with food intake, body weight maintenance, and energy balance (80-82). *Hcrt* encodes hypocretin, a hypothalamic neuropeptide precursor protein that gives rise to orexin neuropeptides that regulate feeding behavior, glucose metabolism, and homeostasis (83). The remaining 2 dysregulated genes—*Mmp11* and *Abhd8*—have not been associated with activities in the brain but have been implicated in diabetes (*Mmp11*) (84, 85) and lipid metabolism (*Abhd8*) (86).

Chronic HFD feeding can lead to diabetes and obesity via activation of proinflammatory pathways in the hypothalamus (7, 8, 87). Whereas the 2 SO diets (SO + CO, PL + CO) resulted in changes in several proinflammatory genes vs the CO diet, the latter diet resulted in the increased expression of just 2 genes linked to inflammation—*Atp2a1* and *Rgs16* (Table 5). *Rgs16* has a prominent role in the trafficking of B and T lymphocytes and macrophages (88) but in the hypothalamus may have a role in circadian regulation and food intake (89). Five of the 9 genes dysregulated by both SO + CO and PL + CO vs CO and associated with both metabolic and neurological disorders are linked to inflammation (*Nr4a2*, *Ghsr*, *Oxt, Dio2*, and *Mt1)*. These genes are different from the ones reported by others: *IKKbeta*, *NF-kB*, and *c-Jun N-terminal kinase* (7, 8), which could be because the diets used in those studies were lard based and contained a mix of saturated and unsaturated fats.

### Impact of soybean oil diet on other genes linked to neurological disease

In addition to being linked to metabolic disorders, OXT signaling is associated with neurological disorders and behavioral and psychiatric functions (6, 90). For example, low levels of OXT in the brain are related to depression (91), schizophrenia (92), pain, and autism (91, 93) and OXT treatment targeting both the central and peripheral systems has been shown to help ameliorate some of these conditions (94, 95). Could low protein levels of OXT observed in the hypothalamus of mice fed the SO diets potentially be linked to neurological dysfunction?

The results of 2 recent studies support the notion that HFD affects some aspect of central OXT signaling and reveal the behavioral implications of such an effect. In the first study, reduced OXT levels in the prefrontal cortex of rats fed an HFD were associated with reduced functionality of OXT signaling, namely impaired social preference, memory, and synaptic potentiation, effects that were normalized by prefrontal injections of an OXT agonist (96). In the second study, a maternal HFD (lard-based, high in PUFAs as well as saturated fats) reduced the density of OXT-immunoreactive neurons in the hypothalamus and negatively affected “OXT-relevant” social behavior in offspring (97). Thus, it is conceivable that SO diets resulting in a decrease in the central actions of OXT could play a role in modulating neurological function and behavior.

In addition to OXT, several other genes associated with neurological diseases were also dysregulated in both the SO diets but not the CO diet (Table 6). The upregulated genes include *Avp*, associated with schizophrenia (98) and depression (99, 100) (Fig. 4C); *Hcrt*, related to anxiety, depression, and pain (101, 102) (Fig. 5B); and *Pcsk1n*, which is predictive of Alzheimer’s disease via association with the amyloid antiaggregant protein, proSAAS (103) (Fig. 2C). In contrast, *Ankk1*, which has a role in dopamine receptor signaling, was downregulated but only in the PL diet (Fig. 3B). Notably, all these changes would lead to impaired neurological function. Finally, as we observed for oxytocin signaling, in addition to changes in gene expression, future studies will need to take into account changes in the neurotransmitter peptide levels and release. For example, *Kcng1*, the potassium voltage-gated channel modifier that is the only gene with a significantly different level of expression between SO + CO and PL + CO (Fig. 2C), belongs to a family of proteins that regulate neurotransmitter release, suggesting that other neurological signaling systems could be implicated, even if transcript levels are not.

The results presented here demonstrating the impact of dietary SO on gene expression in the hypothalamus lead to the provocative suggestion that dietary fat, in general, and SO, in particular, may have an impact on mental as well as metabolic health. The results also clearly indicate that additional studies are needed to determine the effects of both high-LA and low-LA SO on hypothalamic and potentially other brain function and underscore the need for a careful evaluation of the extensive use of SO-based food products, including infant formula, animal feed, and other processed foods.

## Abbreviations

AIN: American Institute of Nutrition
ALA: alpha linolenic acid
Avp: vasopressin
BSA: bovine serum albumin
CO: coconut oil
DEG: differentially expressed genes
ELISA: enzyme-linked immunosorbent assay
GO: Gene Ontology
GTT: glucose tolerance test
HFD: high-fat diet
IHC: immunohistochemistry
LA: linoleic acid
MNC: magnocellular neuroendocrine cell
OXT: oxytocin
PBS: phosphate buffered saline
PCA: Principal Components Analysis
PL: Plenish
Prkcg: Protein Kinase C γ
PUFA: polyunsaturated fatty acid
PVN: paraventricular
qPCR: quantitative polymerase chain reaction
RPKM: reads per kilobase per million
RT: reverse transcriptase
SD: standard deviation
SO: soybean oil
SON: supraoptic
ST: stigmasterol
TGF-β: Transforming Growth Factor beta
Viv: vivarium

## Additional Information

***Data Availability:*** All processed data are available on Figshare (37).
